# Development and evaluation of recombinant E2 protein based IgM capture enzyme-linked immunosorbent assay (ELISA) and double antigen sandwich ELISA for detection of antibodies to Chikungunya virus

**DOI:** 10.1371/journal.pntd.0010829

**Published:** 2022-12-08

**Authors:** Meijun Guo, Shanshan Du, Lijin Lai, Wei Wu, Xiaoxia Huang, Aqian Li, Hao Li, Chuan Li, Qin Wang, Lina Sun, Tiezhu Liu, Tingting Tian, Shiwen Wang, Mifang Liang, Dexin Li, Chun Xie, Jiandong Li

**Affiliations:** 1 School of Public Health, the key Laboratory of Environmental Pollution Monitoring and Disease Control, Ministry of Education, Guizhou Medical University, Guiyang, China; 2 NHC Key Laboratory of Biosafety, China CDC, Beijing, China; NHC Key Laboratory of Medical Virology and Viral Diseases, National Institute for Viral Disease Control and Prevention, China CDC, Beijing, China; 3 Shenzhen Hospital of the University of Chinese Academy of Sciences (Guangming), Shenzhen, China; Instituto Butantan, BRAZIL

## Abstract

**Background:**

Chikungunya virus (CHIKV) reemerged and caused millions of human infections since 2004. The disease could be established, when the virus has been introduced to areas where the appropriate vectors are endemic. The differential diagnosis of CHIKV infection varies based on place of residence, travel history, and exposures. Serological tests are commonly used to diagnose CHIKV infection, but their availability and assessments of the performance of the diagnostics have been limited.

**Objectives:**

To develop and evaluate antibodies detection methods for chikungunya diagnosis and serological investigation.

**Methods:**

Recombinant E2 protein based IgM capture enzyme-linked immunosorbent assay (Mac-ELISA) and double antigen sandwich ELISA (Das-ELISA) for detection of antibodies to Chikungunya virus were developed and evaluated. The repeatability was evaluated by testing of three reference sera at single dilutions in triplicated for 5 times. The sensitivity, specificity, accuracy, and agreement of the MAC-ELISA and Das-ELISA were obtained by comparing the detection results of 225 serum samples (45 positive; 180 negative) with a real-time RT-PCR assay and an IFA commercial tests manufactured by Euroimmun.

**Results:**

The established ELISA assays were standardized by determining the optimal concentrations of the key reagents. The coefficient values of repeat testing were within 10% and 20% for intraassay and interassay precision, respectively. A sensitivity of 60.0% and 52.5%, a specificity of 96.2% and 96.8%, and an accuracy of 89.8% and 88.9% were obtained for the Mac-ELISA and Das-ELISA, respectively, when compared to a CHIKV qRT-PCR method. And a sensitivity of 100%, a specificity of 97.5% and 99.5%, and an accuracy of 97.8% and 99.6% were yielded respectively when using the IIFT as a reference method, which showed a highly consistence to the commercial IIFT assay with a Kappa value greater than 0.90.

**Conclusions:**

The Mac-ELISA and Das-ELISA based on recombinant E2 protein of CHIKV were developed and standardized, which could detect IgM or total antibodies against CHIKV in 2–3 hours with acceptable sensitivities and specificities. These assays can be used for laboratory diagnosis and serological investigation of CHIKV infections to evaluate the risk of CHIKV transmission.

## Author summary

CHIKV reemerged and caused millions of human infections since 2004. The disease could be established, when the virus has been introduced to areas where the appropriate vectors are endemic. Early detection, active response and followed epidemiological evaluation will provide opportunity to prevent CHIKV dissemination. The differential diagnosis of CHIKV infection varies based on place of residence, travel history, and exposures. Serological tests are commonly used to diagnose CHIKV infection, but their availability and assessments of the performance of the diagnostics have been limited. This study reports the development, standardization, and clinical evaluation of a Mac-ELISAs and a Das-ELISA for the diagnosis and surveillance of chikungunya. Comparing the detection results of 225 serum samples (45 positive; 180 negative) with a real-time RT-PCR assay and an IFA commercial tests manufactured by Euroimmun, confirming the usefulness of these ELISA methods. But a limitation of this study is the lack of a gold standard detection method as a reference and the lack of enough clinically confirmed CHIKV infection samples for evaluation. Serological testing for CHIKV needs to be further explored.

## Introduction

Chikungunya virus (CHIKV) belongs to the family *Togaviridae*, genus *Alphavirus* that can cause an acute onset of fever and polyarthralgia [1]. CHIKV has a single-stranded, positive-sense RNA genome, and mainly transmitted to humans by *Aedes aegypti and Aedes albopictus* [1]. The virus was first isolated in 1952 in Tanzania [2], and reemerged in Africa, Asia, and the Indian and Pacific Oceans since 2004, resulting in millions of human infections [3]. The disease could be established, when the virus has been introduced to areas where the appropriate vectors are endemic. In 2008, imported case of CHIK was firstly detected in China [4], and since then, imported cases were often reported, and an outbreak of CHIK through local transmission was occurred in 2010 in Guangdong province, China [5]. In 2013, local transmission of CHIKV was reported in the Caribbean, and then the virus has disseminated rapidly throughout the Americas [6]. So far, more than 100 countries have reported local or imported cases throughout the world. People could transmit CHIKV to a biting mosquito when the patient is at a viremic phase, usually during the first week of illness, which played an important role in the spread of the virus.

The differential diagnosis of CHIKV infection varies based on place of residence, travel history, and exposures. Laboratory diagnosis is generally to detect virus, viral RNA, or virus-specific IgM antibodies in serum or plasma collected within the first 5 days after onset, after which the CHIKV-specific antibody detection assay is a sensitive test [7,8]. A variety of commercially available or in-house used CHIKV antibodies detection assays were reported, but the availability and assessments of the performance have been limited [8,9]. Some were developed based on inactivated CHIKV particle [10], which is generally unfavorable for long-term storage, especially in areas with only sporadic case reports. Serological test reagents with the characteristics of cheap, stable and easy to preserve are needed for the diagnosis and investigation of CHIK in areas with the appropriate vectors for CHIKV transmission.

The E2 protein of CHIKV is a potential serodiagnostic target antigen, which can stimulate the immune response of the infected body and induce high-level specific antibodies [5,11,12]. The capture format of the Mac-ELISA for IgM detection eliminates potential background caused by extraneous antibody, resulting in less-frequent nonspecific reactions and removing false-positive reactions caused by rheumatoid factor [13,14]. Double-antigen sandwich ELISA (Das-ELISA) has been used to detect antibodies against different pathogens successfully, regardless of class and species, which provides great convenience for laboratory detection and field investigation [15,16]. In this study, we produced the extracellular domain of E2 protein using *Eescherichia coli*-based expression systems, developed a Mac-ELISA and a Das-ELISA for the diagnosis and surveillance of chikungunya, which are low cost, good productivity and sable for storage. The two methods showed high sensitivity and specificity to achieve the goals of chikungunya diagnosis and surveillance.

## Materials and methods

### Ethics statement

This study was approved by the Institutional Ethical Committee of National Institute for Viral Infectious Disease Control and Prevention, China CDC (IVDC2019-018). Formal written informed consent was obtained from the patients and healthy adults donors when the serum samples were collected.

### Clinical samples

A total of 45 serum samples from CHIKV infected patients in acute phase (day 1–11 after onset of illness) confirmed by CHIKV real-time reverse-transcription polymerase chain reaction (qRT-PCR) or indirect immunofluorescence test (IIFT) (EUROIMMUN, Lübeck, Germany) were used in this study, 31 sera were collected during an outbreak of CHIK fever in Guangdong province in 2010 [17], 14 sera were collected from CHIK cases imported from abroad since 2008 (4). A total of 180 control sera was assembled from Chinese National Institute for Viral Diseases Control and Prevention serum collections, samples were selected on the basis of having no known infection of CHIKV, including sera from healthy adult donors (n = 70), patients of hantavirus caused hemorrhagic fever with renal syndrome (n = 50), patients of dengue fever (n = 30), SFTSV infected patients (n = 30).

### Production of the CHIKV-rE2 protein

To construct the plasmid for recombinant expression of the extracellular domain of E2 protein, a gene fragment with a length of 1122 nt was amplified by PCR from CHIKV strain SD08Pan, a six-histidine tag coding gene, *Nde* I and *Xho* I restriction sites were introduced at the 5’- and 3’-ends respectively using specific primers, to allow the cloning of the coding gene into pET-30a vector (Novagen, USA). The recombinant plasmid was transformed into E. coli BL21(DE3) PlysS using a heat shock method. Expression was obtained after Isopropyl β-D-1-thiogalactopyranoside (IPTG) induction according to manufacture instruction. Cells were harvested by centrifugation at 8000 g for 20 min and were resuspended in Tris-HCl buffer (50 mM Tris-HCl, pH 7.5, 0.5 mM EDTA, 0.3 M NaCl), ultrasound was performed to break the cells in an ice bath, and then centrifuged and resuspended in a washing buffer (50 mM Tris-HCl, 300 mM NaCl, pH 7.5, 2 M urea) twice, then centrifuged and resuspended in denaturing buffer (50 mM Tris-HCl, 8 M urea, 500 mM NaCl, PH7.5), after stir at room temperature for 1 h, 4°C overnight. The rE2 protein was purified using a HiTrap Protein HP column (GE Healthcare Life Sciences) and following the manufacturer’s instructions. The purified rE2 protein was refolded through stepwise dialysis method against urea in refold buffer (20 mM PBS, 6 M urea, 500 mM NaCl, 500 mM L-arginine, 4 mM reduced glutathione, 0.4 mM oxidized glutathione, PH8.0), this refolding buffer was kept at 4°C for 6–8 h with gentle stirring, and reduce the urea concentration from 4 to 0.2 M, and finally against PBS buffer containing 300 mM NaCl. The final obtained protein was stored at −80°C. The produced rE2 protein was analyzed using a 12% SDS-polyacrylamide gel, electrophoresis separated proteins were detected by Coomassie blue G250 (Biodragon, China) staining. In addition, SDS-PAGE separated proteins were transferred to a cellulose nitrate membrane by semidry blotting. After blocking for 1 h in 5% PBS-M, the filters were incubated with chikungunya virus immunized mouse serum at a dilution of 1:100. For the detection of specific antibody binding, horseradish peroxidase (HRP)-labeled anti-mouse IgG conjugate (Sigma) was used.

### Antigen labeling

The purified rE2 protein was labeled with horseradish peroxidase (HRP) according to published method [12], the commercial HRP powder (sigma, Germany) was dissolved in 100 mM sodium acetate, pH 4.4 at a concentration of 5 mg/ml. Freshly prepared 100 mM sodium periodate (NaIO4) was added to the protein solution in aliquots at 2 minute intervals in the dark to a final concentration of 20 mM, and incubate in the dark for an additional 20 minutes. Separate the oxidized HRP from the NaIO4 by dialysis in 0.1 M carbonate hydrate buffer, pH 9.5. Add 2 mg of the purified rE2 protein per one ml of the activate HRP solution; incubate at 4°C overnight. After addition of 0.1 mL freshly prepared NaB3H4, the mixture was incubated at 4°C for 2 h. And the labeled rE2 protein was separated from unreacted material by gel filtration.

### IgM capture ELISA (Mac-ELISA)

Strips of flat-bottom polystyrene microwells (Nunc Immuno MaxiSorp, ThermoFisher) were coated overnight at 4°C with 100 μL per well of anti-human IgM immunoglobulin at a concentration of 5 μg/mL in carbonate buffer (0.05 M, pH 9.6). Wells were washed three times with phosphate-buffered saline (PBS) containing 0.05% Tween20 (PBS-T) and blocked with 5% skimmed milk powder in PBS-T (PBS-TM) for 30 min at 37°C. Then 100 μL of serum diluted 1: 20 in PBS-T was added and incubated for 1 hour at 37°C. After five washes with PBS-T, 100 μL of HRP-conjugated CHIKV rE2 antigen (2 μg/ml) in PBS-TM was added and incubated for 30 min at 37°C. After five washes with PBS-T, 50 μl of substrate tetramethylbenzidine (TMB) (Calbiochem) was added for 10 min at room temperature, and the reaction was terminated by the addition of 50 μL of 2 M sulfuric acid. The optical density (OD) at 450 nm was measured in a DTX 880 multi-mode detector (Beckman Coulter, CA, USA) with incidence wavelength 450 nm and reference wavelength 620 nm. Positive and negative controls consisted of serum samples with known anti-CHIKV IgM responses that tested negative for Dengue virus, and Zika virus. The OD value from negative control wells should be less than 0.1. Samples with an OD higher than the average of the negative controls plus 3 standard deviations were considered positive.

### Double-antigen sandwich ELISA (Das-ELISA)

Strips of flat-bottom polystyrene microwells (Nunc Immuno MaxiSorp, ThermoFisher) were coated overnight at 4°C with 100 μL per well of purified rE2 Protein at a concentration of 4 μg/mL in carbonate buffer (0.05 M, pH 9.6). Wells were washed three times with PBS-T and blocked with PBS-TM for 30 min at 37°C. Then 50 μL of serum diluted 1:10 in PBS-TM was added, followed by addition of 50 μl of HRP-conjugated CHIKV rE2 antigen (2 μg/ml) diluted in PBS-TM, and incubated for 1 hour at 37°C. After five washes with PBS-T, 50 μL of substrate TMB was added for 10 min at room temperature, and the reaction was terminated by the addition of 50 μL of 2 M sulfuric acid. The optical density (OD) at 450 nm was measured in a DTX 880 multi-mode detector (Beckman Coulter, CA, USA) with incidence wavelength 450 nm and reference wavelength 620 nm. Samples with an OD higher than the average of the negative controls plus 3 standard deviations were considered positive. Positive and negative controls consisted of serum samples with known anti-CHIKV IgG responses that tested negative for Dengue virus, and Zika virus.

### Indirect immunofluorescence test

Indirect immunofluorescence test (IIFT) (EUROIMMUN, Lübeck, Germany) was used to detect the CHIKV-specific antibodies in the sera collected from suspected patients according to manufacturers instruction. In short, the serum samples were diluted 1:10–1:80, and 25 μL of the dilutes were applied to the reaction fields of the slides, and then incubated for 1 h. Rheumatic factor was pre-adsorbed with EUROSORB reagent for the detection of IgM antibodies. Anti-human IgG or IgM antibodies labeled with fluorescein isothiocyanate (FITC) were diluted and loaded onto the slides. The fluorescent cells were observed under an Olympus fluorescence microscopy.

### Statistical analysis

The sensitivity, specificity, accuracy, and their 95% Confidence Intervals (95% CI), as well as kappa indices, were calculated using the statistical package of Microsoft Excel 2013, GraphPad Prism (Version 5 for Windows, San Diego, CA, USA), and IBM SPSS Statistics version 22. Chi-Square (Χ^2^) tests were performed for association of the comparative analysis with SPSS, when p value≤ 0.05, the results were accepted as a level of significance.

## Result

### Preparation of recombinant CHIKV rE2 protein

**Fig 1:**
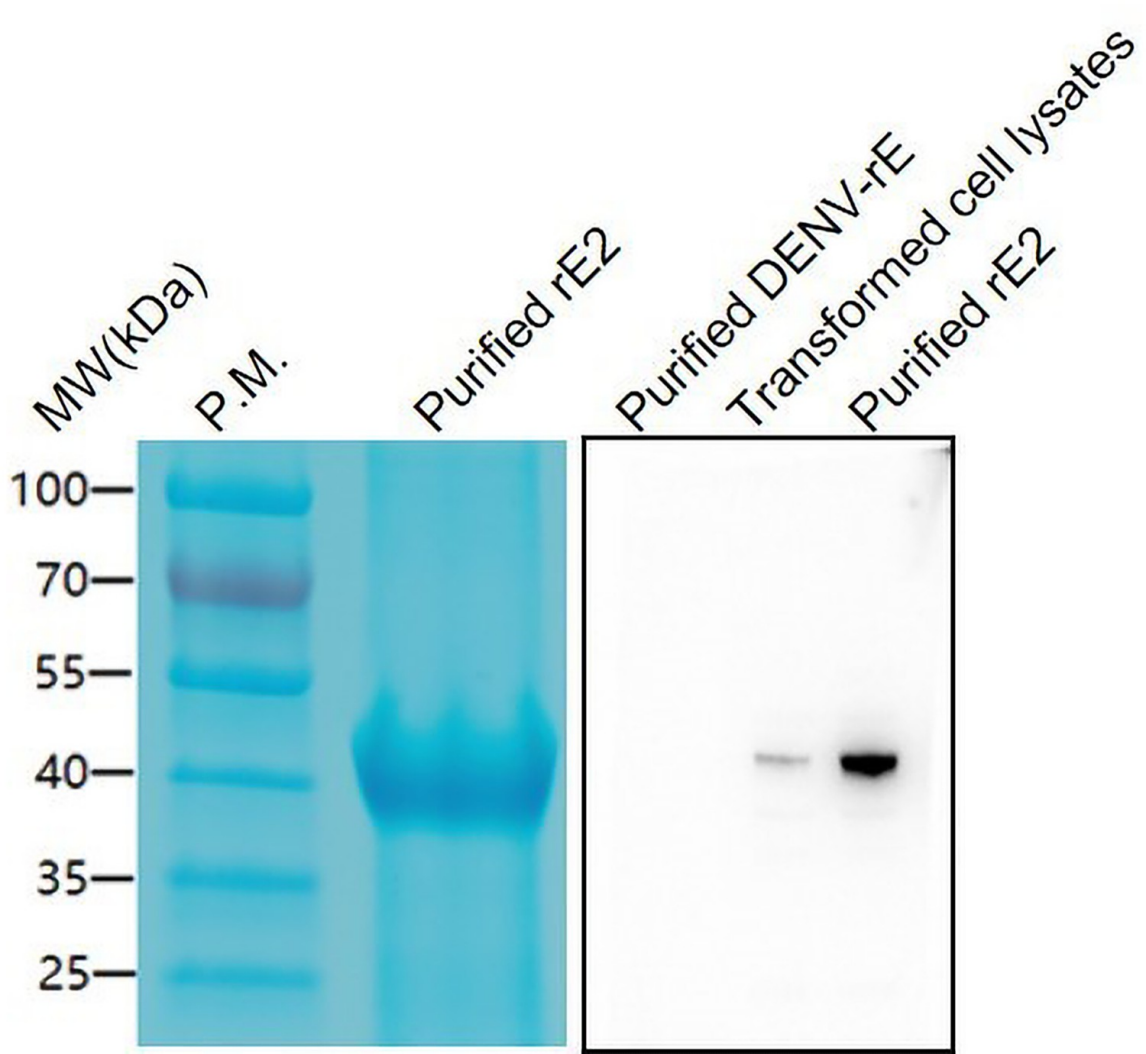
SDS-PAGE and immunoblot analysis of CHIKV rE2 protein produced in E. coli. MW(kDa), Molecular weight (kilodaltons); P.M., Prestained protein marker; purified rE2, recombinant CHIKV rE2 protein after purification by Ni-NTA column chromatography; purified DENV-rE, purified recombinant extracellular domain of E protein from dengue virus type 2; Transformed cell lysates, BL21 DE3 E. coli cells were transformed with pET-30a-CHIKV-rE2 plasmid without inducing with IPTG.

The recombinant produced the extracellular domain of E2 proteins (rE2) were detected in Coomassie blue-stained SDS-PAGE (Fig 1) with a molecular weight about 40 kDa. In the immunoblot, recombinant rE2 proteins of the expected molecular weights were recognized by CHIKV polyclonal antibodies, while no blots were found in the lane of the purified recombinant extracellular domain of E protein from dengue virus type 2, which indicated that the purified and unpurified, His-tagged rE2 proteins reacted with CHIKV immunized mouse serum (Fig 1).

### Development of the ELISA methods

**Fig 2:**
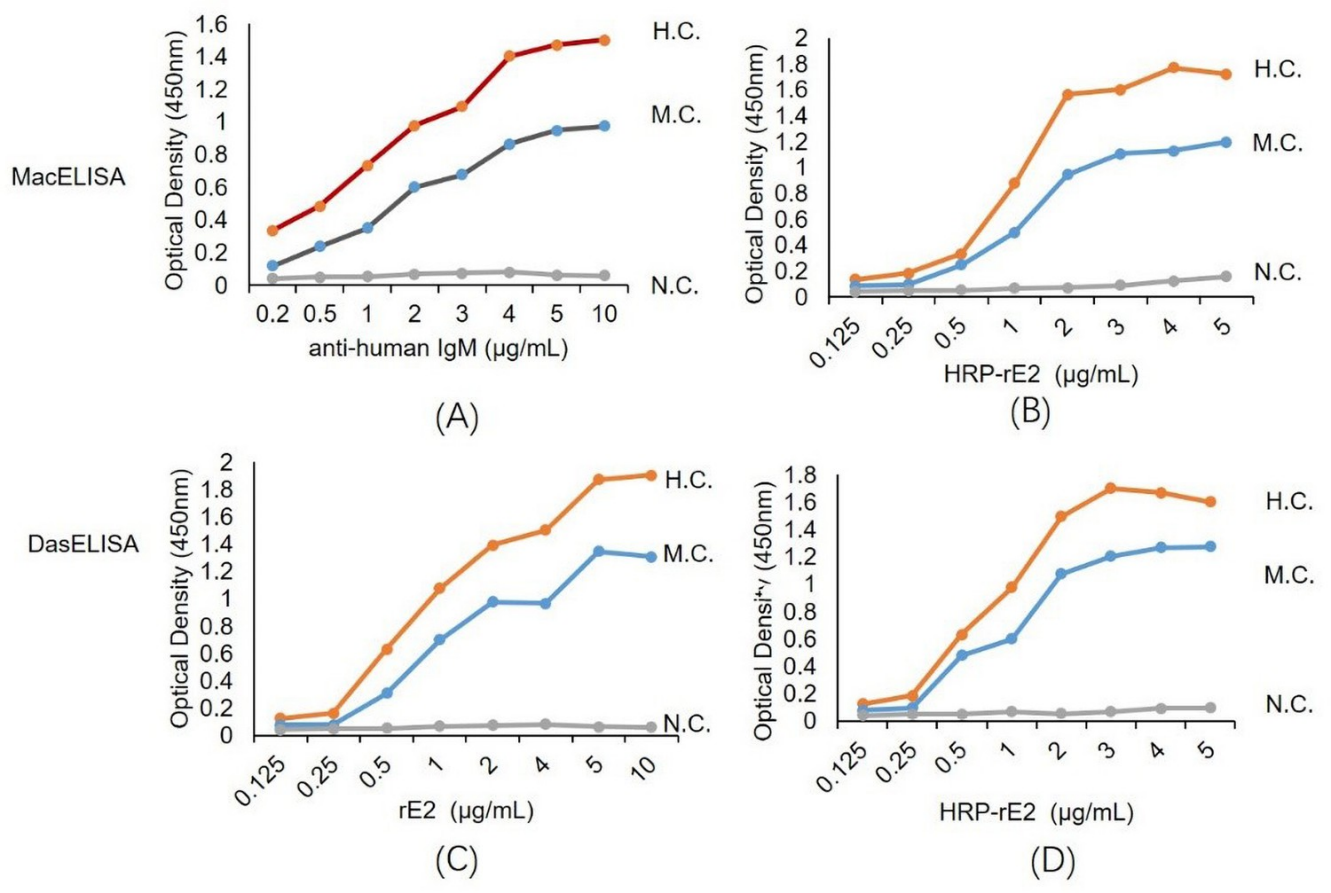
Determination of the optimal concentrations of key components of the Mac-ELISA and Das-ELISA.

The optimal concentrations of the capture antibody, antigen, and detection antigen conjugate used in the established ELISA methods are determined through square titration curve using patient serum positive and negative controls (Fig 2). To determine the precision (repeatability) of ELISAs, two levels of positive controls, were tested in duplicate at single dilutions selected to represent high, and medium OD value in the ELISA tests (Fig 2). It was used as the criteria to select dilutions that could generate OD values > 1 for high positive controls, ≈1 for medium positive controls, and < 0.1 for negative controls, respectively. The optimal capture antibody concentrations and the detection antigen conjugate of CHIKV rE2-HRP were 5 μg/ml and 3 μg/ml respectively for MAC-ELISA (Fig 2A and 2B), and similarly, the optimal capture concentration of CHIKV rE2 and the detection antigen conjugate of rE2-HRP were 5 μg/ml and 3 μg/ml respectively for Das-ELISA (Fig 2C and 2D), as no significant increase in the OD of the positive controls was detected at higher concentrations (Fig 2). Using the same criteria, the optimal dilutions for the indirect IgG ELISA were 5 μg/ml for the coating rE2 antigen at Minisorp microwells, and 1:1000 for the commercial HRP-conjugated anti-human antibody, respectively.

### Repeatability of the ELISAs

To determine the repeatability of ELISAs, two positive serum controls and one negative serum controls were tested in triplicate at single dilutions for 5 times. Calculated coefficient of variation (CV) of the intraassay and interassay are given in Table 1. These values are within the accepted values of 10% and 20% for intraassay and interassay precision, respectively [18].

**Table 1:** Repeatability estimates for indirect IgG ELISA and MacELISA.

	MacELSIA		DasELISA	
	CV(%)*	Range	CV(%)	Range
Intra-assay				
HC	6.25±3.05	(2.70–11.38)	6.52±1.83	(0.85–10.05)
MC	8.73±1.98	(3.36–14.25)	8.06±2.15	(1.24–12.05)
NC	4.5±1.59	(2.46–8.68)	3.8±1.48	(0.96–6.68)
Inter-assay				
HC	9.25±2.78	(1.7–14.6)	9.69±2.88	(5.05–14.66)
MC	11.82±3.35	(3.48–16.57)	12.28±3.15	(6.55–17.74)
NC	6.57±2.58	(3.46–13.06)	5.86±2.48	(3.96–12.63)

* Repeatability estimates for high positive serum control (HC), medium positive serum control (MC) and negative serum control (NC) were calculated as the percent coefficient of variation (CV = standard deviation of replicates/mean of replicates×100).

### Sensitivity, specificity and accuracy

The sensitivity was evaluated by using 45 sera collected from CHIKV infected patients at acute phage (1–11 days post symptom onset) confirmed by a real-time reverse transcription PCR (qRT-PCR), and an IIFT assay (Table 2). Results showed that serum samples collected within 24 hours post onset of illness, no antibodies were detected. For the serum samples collected at day 1–3, the antibodies could be detected by MacELISA (7/19, 36.8%), DAS-ELISA (5/19, 26.32%), and indirect IgG ELISA (4/19, 21.05%) as well as the reference method of IIFT (4/19, 21.05%) (Table 2). The antibody detection rate increased significantly among the serum samples collected during 4–6 days after onset of illness (Table 2). As to 7–11 days after onset of illness, antibodies could be detected from all the collected 8 serum samples, the detection rate reached 100%, however, the viral RNA detection rate by qRT-PCR decreased significantly (Table 2). The differences of antibodies detection rate were not statistically significant (X^2^ = 0.622, P>0.05).

**Table 2:** CHIKV specific antibodies detection in the sera of laboratory diagnosed patients*.

Days post symptom onset (Nr. of samples collected)	Nr. of detected (%)				
	MacE LISA	Das-ELISA	IIFT-IgM	IIFT-IgG	qRT-PCR
<1(n = 3)	0 (0%)	0 (0%)	0 (0%)	0 (0%)	3 (100%)
1~3(n = 19)	7 (36.8%)	5(26.3%)	5 (26.3%)	4 (21.1%)	19 (100%)
4~6(n = 15)	14 (93.3%)	13 (86.7%)	13 (86.7%)	13 (86.7%)	15 (100%)
7~11(n = 8)	8 (100%)	8(100%)	8 (100%)	8 (100%)	3 (37.5%)
Total (n = 45)	29 (64.4%)	26 (57.8%)	26 (57.8%)	26 (57.8%)	40 (88.9%)

* Chi-Square (Χ^2^) tests were performed for the comparative analysis with SPSS, when p value≤ 0.05, the differences were accepted as a levels of statistical significance.

When the panel of control serum samples (n = 180) was tested, the numbers of true negatives and false positives were 178 and 2 for MacELISA detection, 179 and 1 for Das-ELISA detection, 176 and 4 for indirect IgG ELISA detection respectively (Table 3). Sensitivity, specificity and accuracy for the established methods were calculated (Tables 3–5). MacELISA and DasELISA yielded a sensitivity of 100%, the specificity ranged from 97.5% (MacELISA) to 99.5% (DasELISA), and their accuracies were 97.8% and 99.6% respectively when using the IIFT as a reference (Tables 4 and 5). Moreover, the kappa index of the established MacELISA (Kappa value = 0.90) and DasELISA (kappa value = 0.98) demonstrated a high consistency with the IIFT. When compared to CHIKV qRT-PCR method, it might be due to the fact that the positive samples used for comparison were mainly collected from CHIK patients at the acute phase, the sensitivity was about 60.0% (MacELISA) and 52.5% (DasELISA), the specificity was about 96.2% and 96.8%, and the accuracies were 89.8% and 88.9% for MacELISA and DasELISA respectively (Tables 4 and 5). The kappa index of MacELISA (kappa value = 0.62) and 0.56 for DasELISA (kappa value = 0.56), which showed a good agreement with the CHIKV qRT-PCR (Tables 4 and 5).

**Table 3:** Antibody detection in serum samples using Mac-ELISA and Das-ELISA.

Serum samples	Nr. of negative detection (%)	
	MacE LISA	Das-ELISA
Healthy adults (n = 70)	69 (98.6%)	70 (100%)
HFRS (n = 50)	50 (100%)	50 (100%)
Dengue fever (n = 30)	29 (96.7%)	29 (96.7%)
SFTS (n = 30)	30 (100%)	30 (100%)
Total (n = 180)	178 (98.9%)	179 (99.4%)

**Table 4:** Comparison of Mac-ELISA and Das-ELISA to qRT-PCR and IIFT in laboratory detection of CHIKV infection.

		qRT-PCR		IIFT	
		Positive	Negative	Positive	Negative
MacELISA	Positive	24	7	26	5
	Negative	16	178	0	194
DasELISA	Positive	21	6	26	1
	Negative	19	179	0	198

**Table 5:** Performance of the Mac-ELISA and Das-ELISA compared to the qRT-PCR and IIFT methods in laboratory detection of CHIKV infection.

	Method	Sensitivity (95%CI)	Specificity (95%CI)	Accuracies (95%CI)	Kappa index
Reference IIFT	Mac-ELISA	100(84.8–100)	97.5(94.1–99.1)	97.8(94.8–99.2)	0.90
	Das-ELISA	100(84.8–100)	99.5(97.0–100)	99.6(97.3–100)	0.98
Reference qRT-PCR	Mac-ELISA	60(44.6–73.7)	96.3(92.3–98.3)	89.8(85.1–93.2)	0.62
	Das-ELISA	52.5(37.5–67.0)	96.8(93.0–98.7)	88.9(84.1–92.4)	0.56

## Discussion

Chikungunya virus (CHIKV) reemerged in Asia and Oceania in 2004, and has caused large outbreaks, resulting in millions of human infections [3]. The spread of CHIKV across the international border was mainly caused by travelers who had visited affected areas. When the virus is introduced to areas where the appropriate vectors are endemic, the disease could be established. Early detection, immediate response, and followed epidemiological evaluation will provide the best opportunity to prevent the potential establishment of the CHIKV in a new area. Limited by the short viremia duration, RT-PCR based viral RNA detection methods are only applicable for samples obtained at acute phase of the illness, usually less than 5 days after onset of illness, serological methods are therefore important and highly demanded for case finding and risk assessment [8,9].

ELISAs for detection of virus specific antibodies have a number of advantages compared to other traditional serological tests, such as immunofluorescence assay and virus neutralization tests. The preparation of suitable antigen is one of the factors to hinder the application of ELISAs. Inactivated complete CHIKV particle or recombinant virus like particle based Mac-ELISA assays showed a good performance for IgM antibody detection, however, the preparation of whole viral antigen is difficult and expensive [19,20]. The E2 protein of CHIKV has been proved to be acceptable as diagnostic reagents [21–23], although it lost some antigenic properties compared to the whole virus particle. And, the conjugation of HRP to proteins with ketone reagents does not exert apparent adverse impact to the enzyme activity and protein antigenicity, which was broadly used in many kinds of immunoassay [24]. Das-ELISA is not limited to the detection of class-specific antibodies, which has been shown to offer increased sensitivity [25].

This study reports the development, standardization, and clinical evaluation of a Mac-ELISAs and a Das-ELISA for the diagnosis and surveillance of chikungunya. We used 6 histidine amino acids tagged extracellular domain protein of E2 protein at the carboxyl termini, which leads to highly purified antigen produced from *E*.*coli* with high yield levels. The use of viral rE2 protein antigen conjugates as indicator leads to a simpler and rapid operating procedure. In order to evaluate the ELISAs, 45 serum samples collected from patients of CHIKV infection in acute phase of illness and 180 control serum samples were processed using both Mac-ELISA and Das-ELISA, and compared to the real-time qRT-PCR method and an IIFT assay as the reference methods. The performance characteristics of the ELISAs were evaluated using serum controls of known infection which represented high and medium OD value in the ELISA tests. The coefficient of variation (CV) of intraassay and interassay were within 10% and 20% respectively, which were accepted precision for ELISA assay as diagnostics [13]. A sensitivity of 100%, a specificity of 97.5% and 99.5%, and an accuracy of 97.8% and 99.6% were yielded respectively when using the IIFT as a reference method. However, a sensitivity of 60.0% and 52.5%, a specificity of 96.2% and 96.8%, and an accuracy of 89.8% and 88.9% were obtained for the Mac-ELISA and Das-ELISA, respectively, when compared to a CHIKV qRT-PCR method. These results are consistent with results reported for similar assays such as ELISA-based methods and IIFT commercial tests manufactured by Euroimmun (Lübeck, Germany) [9].

In this study, the serum samples obtained for evaluation were all from patients in acute phase (day 1–11 after onset of illness), IgM and total antibodies were detected by Mac-ELISA and Das-ELISA respectively from less than 30% (7/22) serum samples collected within 3 days after onset of illness, while the detection rate of qRT-PCR was 100%. Among the sera collected 7 days post symptom onset, antibodies detection rate reached 100% (8/8), while the qRT-PCR detection rate decreased to 37.5% (3/8). It was reported that in humans, viremia phase of CHIKV in serum was fairly short, viral RNA sequences were seldom detected in serum collected 7 days after the onset of symptoms [26,27]. IgM were usually detected within 5–7 days after the onset of symptoms, and began to wane over several months after infection [28,29]. IgG antibodies could be detected approximately 7–10 days after onset of illness, often after viremia has been cleared [29]. The disparity between the established ELISA methods and qRT-PCR was probably due to the fact that the versatility of host immune response leading to a kinetic variation of antibodies appearance in serum. Therefore, although IgM antibodies start to develop on day 2 of CHIKV infection, viral RNA based methods, such as qRT-PCR, should be recommended as the preferred detection method for acute-phase samples. A negative Mac-ELISA result may reflect an insufficient antibody response in infection rather than no infection. However, the value of IgM detection for rapid diagnosis should not be neglected, since rapid diagnosis of CHIK is crucial for proper case management and care. As previous studies have demonstrated that viral RNA or antibody-based serological tests can be used to diagnose CHIKV reliably, depending on the time of sample collection. CHIKV RNA or antigen detection tests could serve as a preferred method for samples collected during the acute phase (≤7 days post symptom onset) of CHIKV infection. Likewise, antibodies detection tests should be considered a priority for samples collected in the convalescent phase (>7 days post symptom onset) [9].

One limitation of this study is the lack of a gold standard detection method as a reference, another is the lack of enough clinically confirmed CHIKV infection samples for evaluation, especially serum samples collected at convalescent phase. So far, indigenous transmission of CHIKV was not established in China, serum samples used for the established ELISA methods evaluation were collected from cases occasionally imported into China since 2008 [4], and cases diagnosed in the outbreak of CHIK fever in Guangdong province in 2010 [17], which made it difficult to collect serum from patients at convalescent phase. Even though there are some limitations in this study, the results showed a highly consistence to the commercial IIFT assay with a Kappa value greater than 0.90, which confirms the usefulness of these ELISA methods.

In conclusion, Mac-ELISA and Das-ELISA based on recombinant E2 protein of CHIKV were developed, which could detect IgM or total antibodies against CHIKV in 2–3 hours with acceptable sensitivities and specificities. These assays can be used for laboratory diagnosis and seroprevalence investigation for evaluation the epidemic situation of chikungunya.
